# Supplementary material for: Topoarchitected polymer networks expand the space of material properties
Source: Nat Commun. 2022 Mar 25;13:1622. doi: 10.1038/s41467-022-29245-0 (PMC8956700; doi:10.1038/s41467-022-29245-0)
Supplement: Supplementary file 1 — Supplementary Information [file 41467_2022_29245_MOESM1_ESM.pdf]

Supplementary Information

Topoarchitected polymer networks expand the space of material properties

Xiao Liu[1], Jingping Wu[1], Keke Qiao[1], Guohan Liu[1], Zhengjin Wang[1], Tongqing Lu[1], Zhigang Suo[2*], and Jian Hu[1*]

[1]State Key Lab for Strength and Vibration of Mechanical Structures, International Center for Applied Mechanics, Department of Engineering Mechanics, Xi'an Jiaotong University, Xi'an 710049, China

[2]John A. Paulson School of Engineering and Applied Sciences, Kavli Institute for Bionano Science and Technology, Harvard University, Cambridge, Massachusetts 02138, United States

Email: hujian@mail.xjtu.edu.cn, suo@seas.harvard.edu

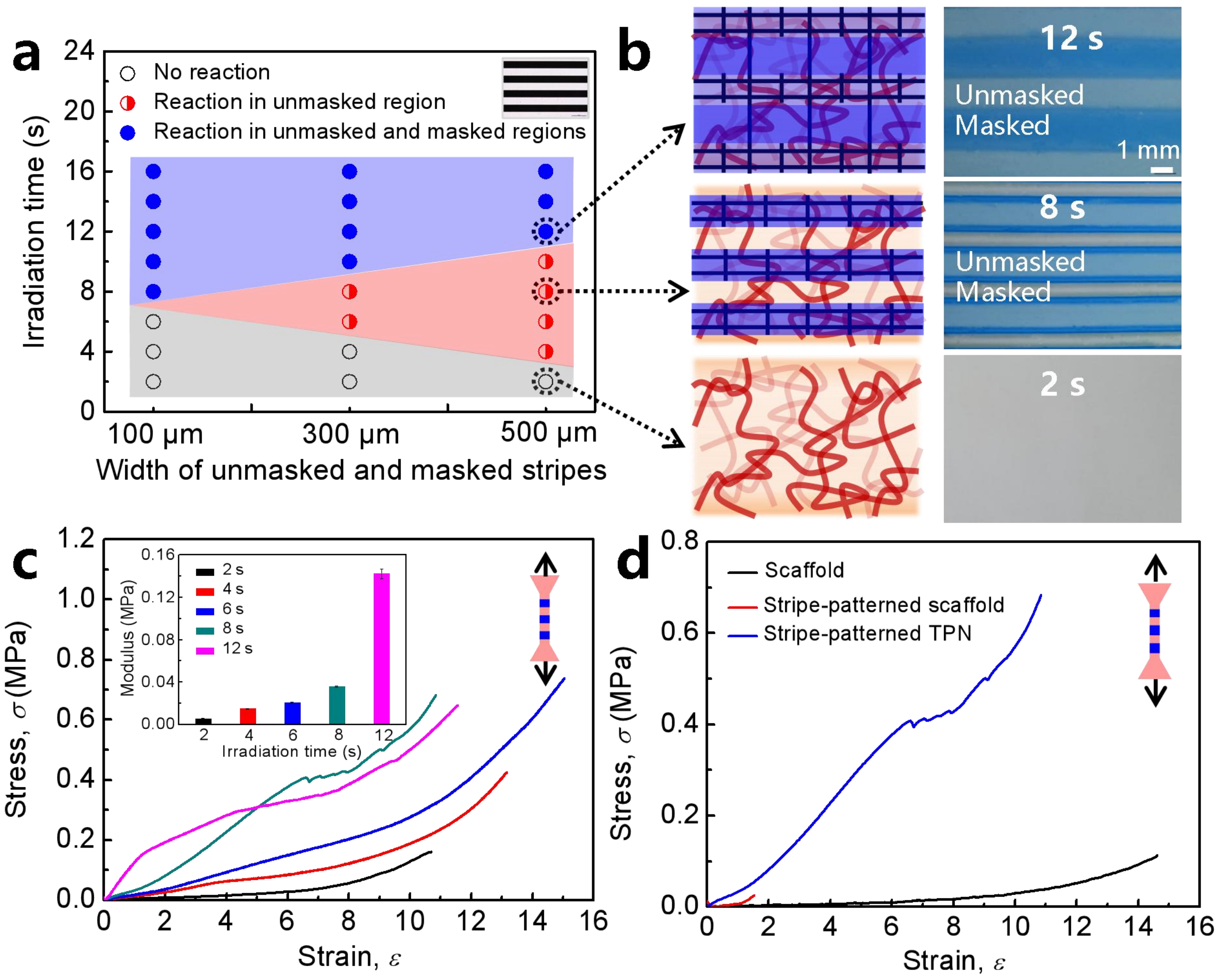

Supplementary Figure 1. Process conditions of stripe-patterned TPNs. a, The morphology of the second network varies with the irradiation time and stripe width. The inset is a micrograph of a mask with alternating unmasked and masked stripes of 500 μm in width. On the plane of the irradiation time and stripe width are three zones. In the gray zone, the second network does not form. In the blue zone, the second network forms in both masked and unmasked region. In the red zone, the second network only forms in the unmasked region. **b,** Schematics and micrographs of the hydrogels. The samples are prepared at different irradiation time, using the mask with the stripe width of 500 μm. **c,** For the hydrogels prepared with various irradiation times, stress-strain curves are measured in the tensile direction vertical to the stripes. The modulus shows an abrupt increase from 0.04 MPa to 0.14 MPa when the irradiation time varies from 8 s to 12 s, due to the formation of the second network in the masked region. At least three samples were tested for the calculation of average value and standard deviation. **d,** Stress-strain curves of the hydrogels at the three stages shown in Fig. 1e.

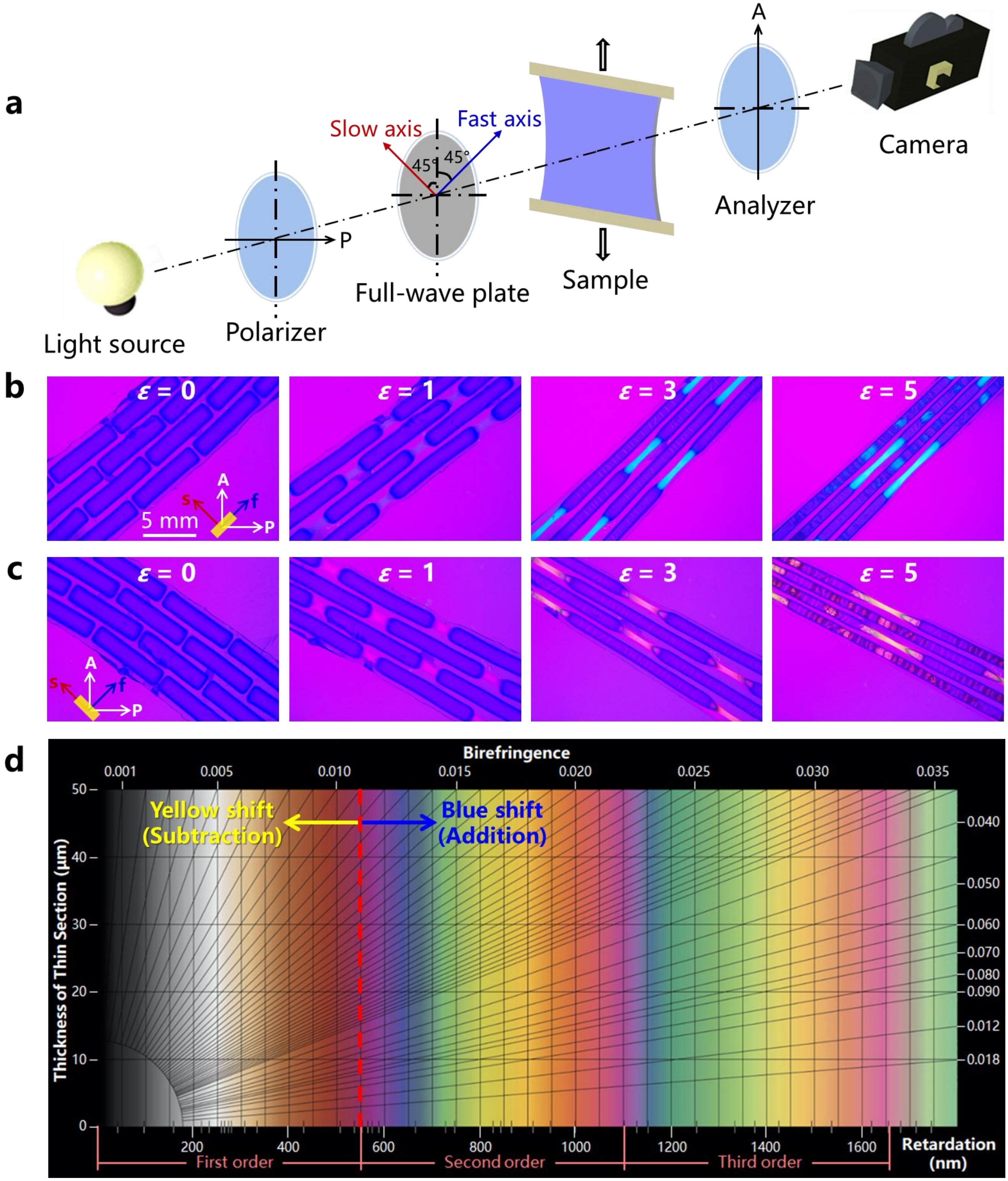

Supplementary Figure 2. Birefringence mechanism of stretched TPNs under polarizing optical microscope. a, Schematic for the configuration of polarizing optical microscope, equipped with the crossed polarizers and a 45° aligned 560 nm full-wave plate. **b, c,** In-situ polarizing optical observation of TPNs with the tensile direction parallel (**b**) or perpendicular (**c**) to the fast axis of the full-wave plate.

The micrographs show blue (**b**) or yellow (**c**) shift with the increase of strain, due to the orientation-induced enhanced birefringence. A: Analyzer; P: Polarizer; s: slow axis of the full-wave plate; f: fast axis of the full-wave plate. **d,** Michel-Levy interference color chart issued by Zeiss Microscopy. The pink background color with 560 nm retardation (highlighted by the red dashed line) shifts to blue when the fast axis of the sample and the full-wave plate are parallel (retardation addition), but shifts to yellow when perpendicular (retardation subtraction). The fast axis of the PAAm matrix, where color change occurs, is along the tensile direction, because PAAm gels have a negative birefringence[1].

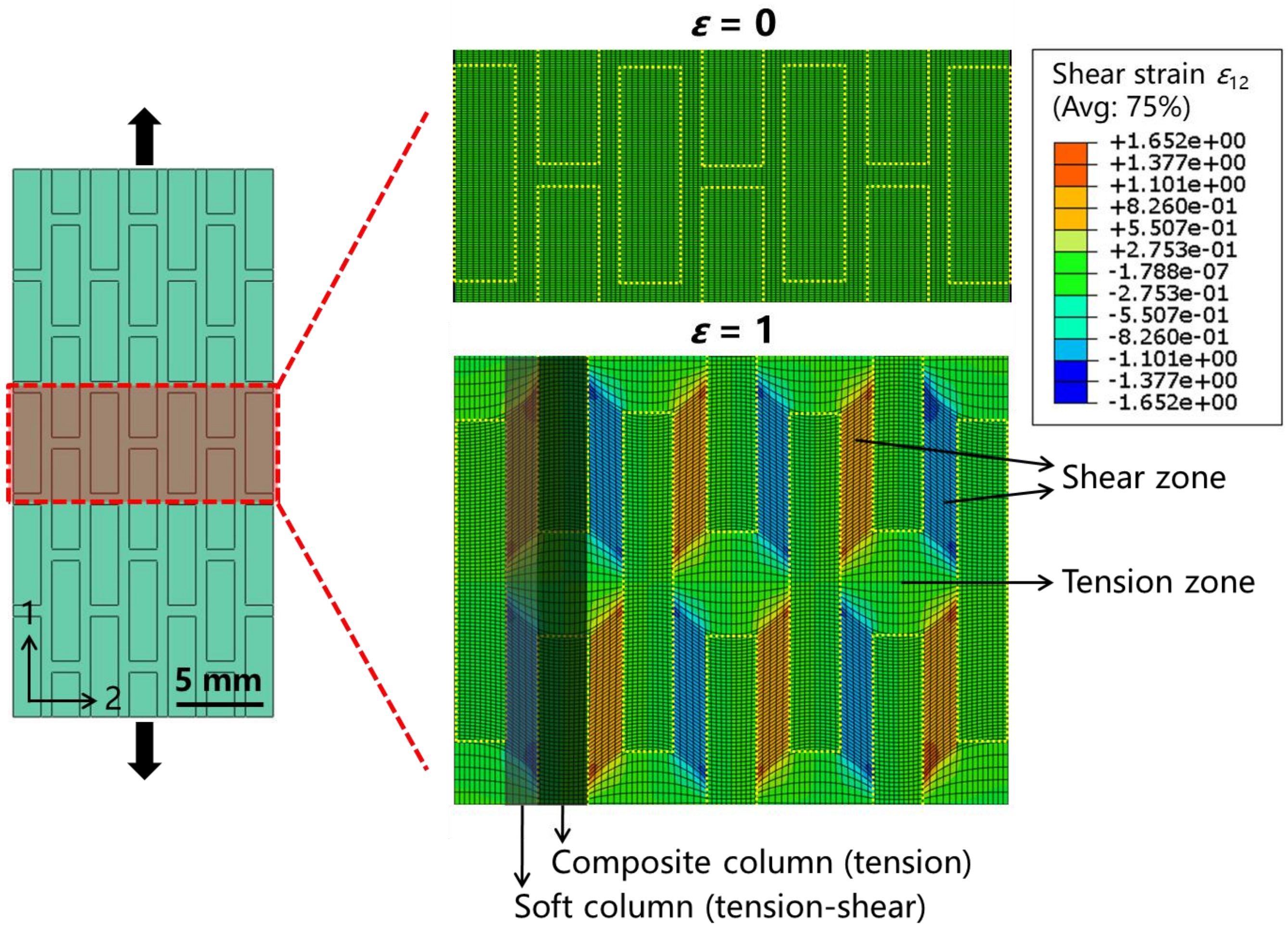

Supplementary Figure 3. Finite element simulation for the shear strain distribution during the tension of TPNs. When the sample is stretched to $\varepsilon = 1$, the strain distribution shows complex tension-shear coupling strain in the soft column and pure tensile strain in the composite column.

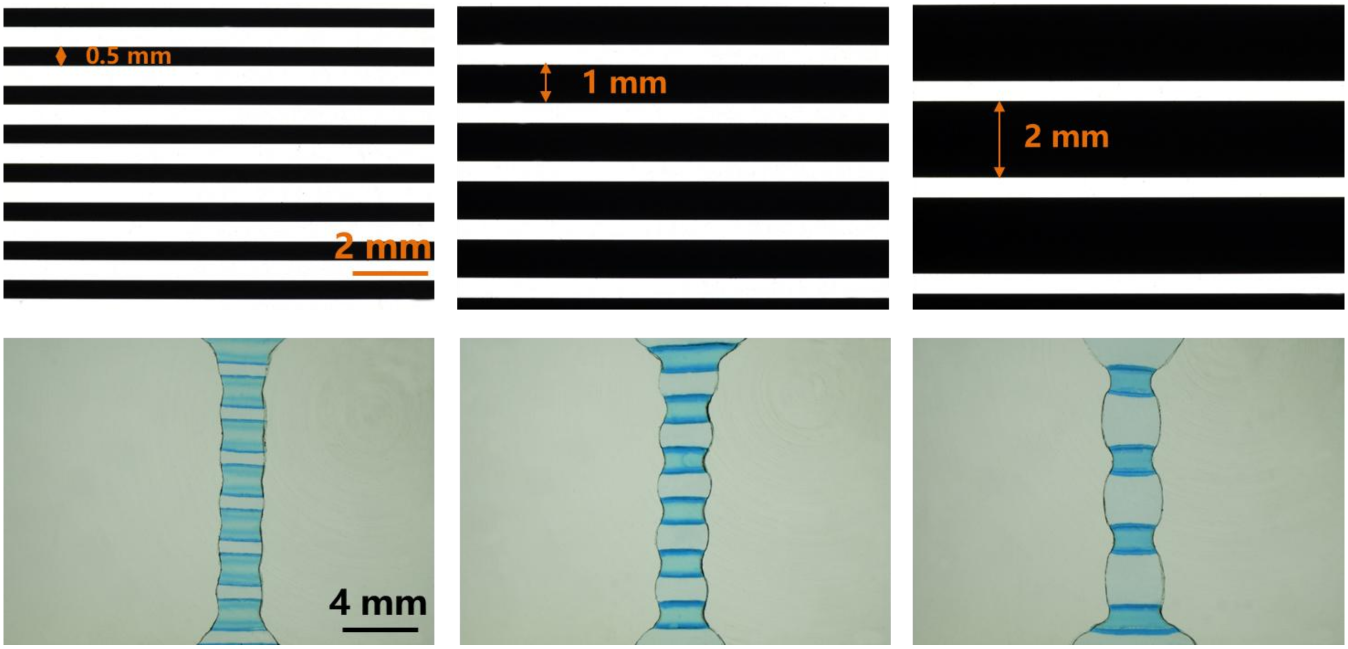

Supplementary Figure 4. Effect of the interval between two neighboring hard phase of stripe-patterned TPNs on the swelling of the soft phase. As the increase in the width of masked stripe from 0.5 mm to 2 mm and fixing the width of unmasked stripe at 500 μm, the hard phase loses the ability to restrict the swelling of the soft phase, which is evidenced by the expanded volume of the soft phase.

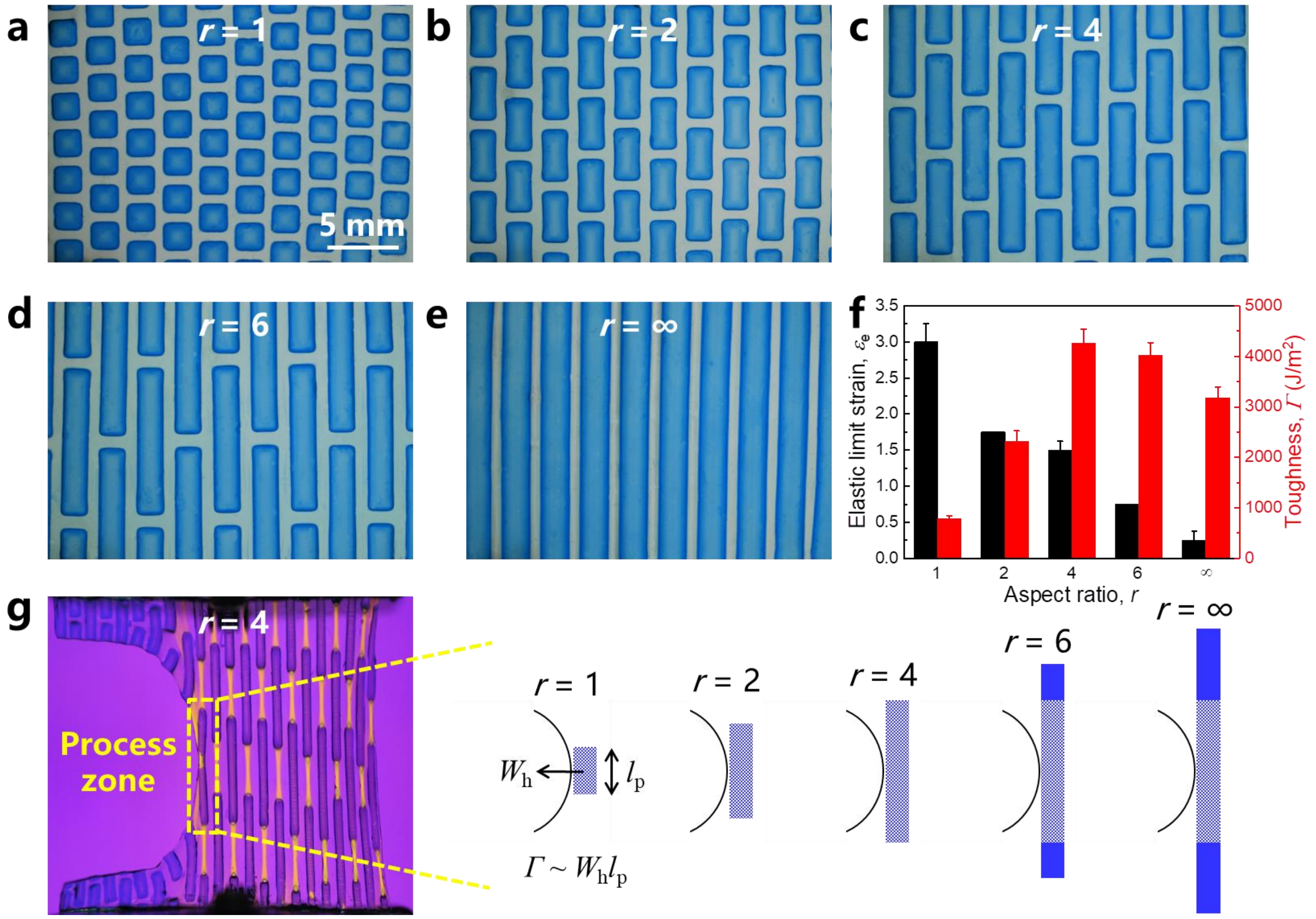

Supplementary Figure 5. Effect of the aspect ratio of the hard phase on the mechanical properties of TPNs. a-e, Optical micrographs of TPNs with varied aspect ratio r of the hard phase. For simplicity, we only change the length of the hard phase and keep the other geometric parameters constant. **f,** Effect of the aspect ratio on the elastic limit strain and toughness of TPNs. At least three samples were tested for the calculation of average value and standard deviation. **g,** Process zone localized in the hard block at the crack front. The process zone size l_p initially increases as the aspect ratio r and then is saturated after exceeding a certain critical r. The toughness Γ of TPNs is mainly determined by the energy dissipation in the process zone. Write $\Gamma \sim W_h l_p$, where W_h is the work of extension of the hard block and can be measured from the tensile stress-strain curve of the hard gel with the same chemical composition.

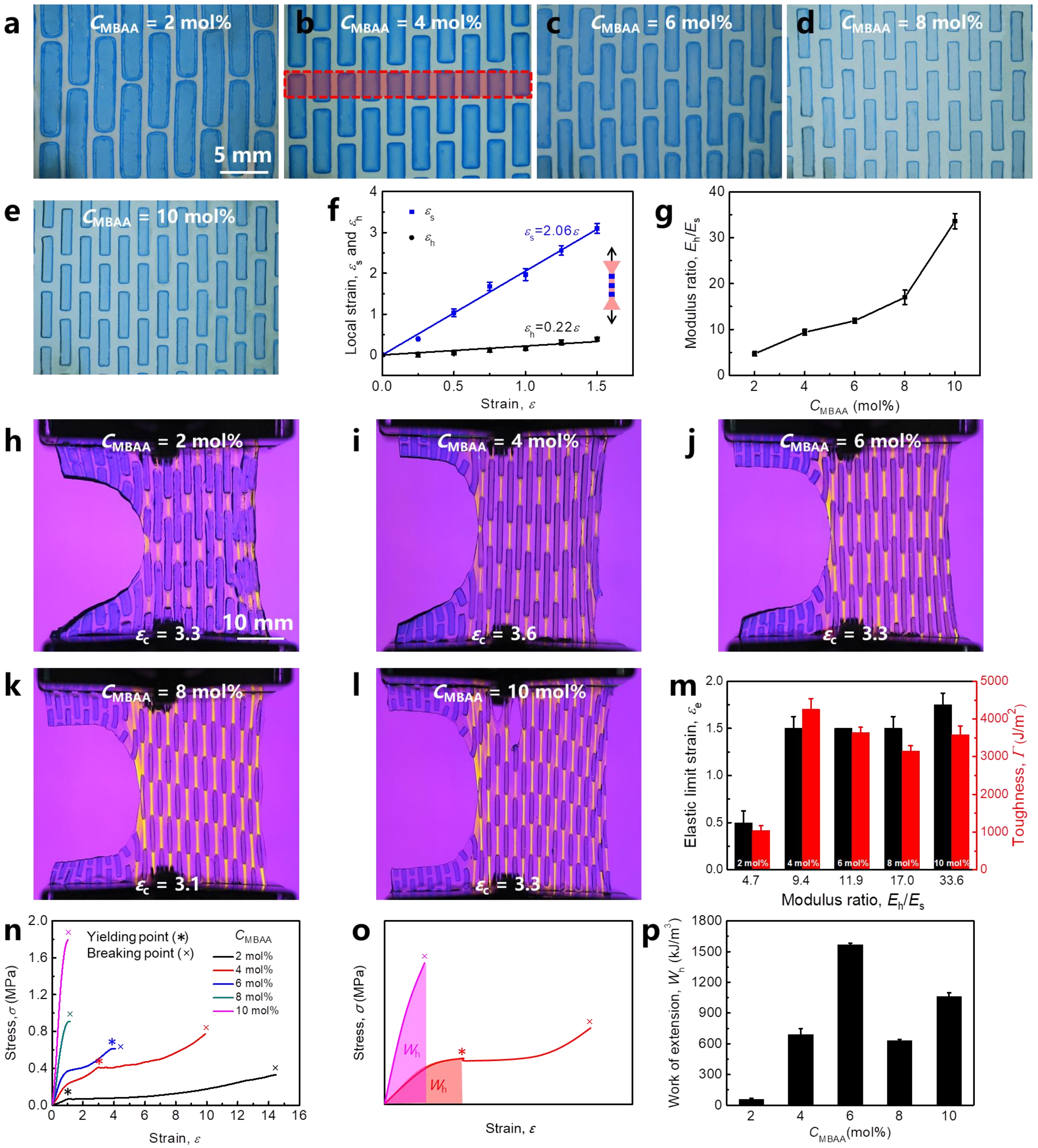

Supplementary Figure 6. Effect of the modulus ratio on the mechanical properties of TPNs. a-e, Optical micrographs of TPNs with varied crosslinker concentration C_{MBAA} for the second network. **f,** Strain ε_s and ε_h of the soft and hard phases in a stripe-patterned TPN as a function of the applied strain ε in the linear elastic region. The stripe-patterned TPN is obtained by cutting the overlap region of the staggered-patterned TPN, marked by the red box in (b). The tensile process of the stripe-patterned TPN

obeys series model (isostress model), so the modulus ratio of the hard phase to the soft phase E_h/E_s = $\varepsilon_s/\varepsilon_h$ = k_s/k_h, here k means the slope of the fitting line. **g,** Dependence of E_h/E_s on C_{MBAA}. **h-i,** In-situ polarizing optical observation of various precut TPNs at the critical strain ε_c. **m,** Effect of E_h/E_s on the elastic limit strain and toughness of TPNs. At least three samples were tested for the calculation of average value and standard deviation. **n,** Stress-strain curves of the hard gels with varied C_{MBAA} to approximatively reflect the mechanical behaviors of the hard phase in TPNs. As the increase of C_{MBAA}, the hard gels show two types of stress-strain response, with yielding (2 mol%, 4 mol%, and 6 mol%) and without yielding (8 mol% and 10 mol%). **o,** Work of extension W_h of the hard block at the crack front of TPNs estimated by integrating the stress-strain curves of the hard gels. In the case with yielding, W_h is estimated by integrating to the yielding point, because the hard block at the crack front once yields, will break immediately without a whole yielding process present in the tension of the hard gel. In the case without yielding, W_h is estimated by integrating to the breaking point. **p,** Dependence of W_h on C_{MBAA}.

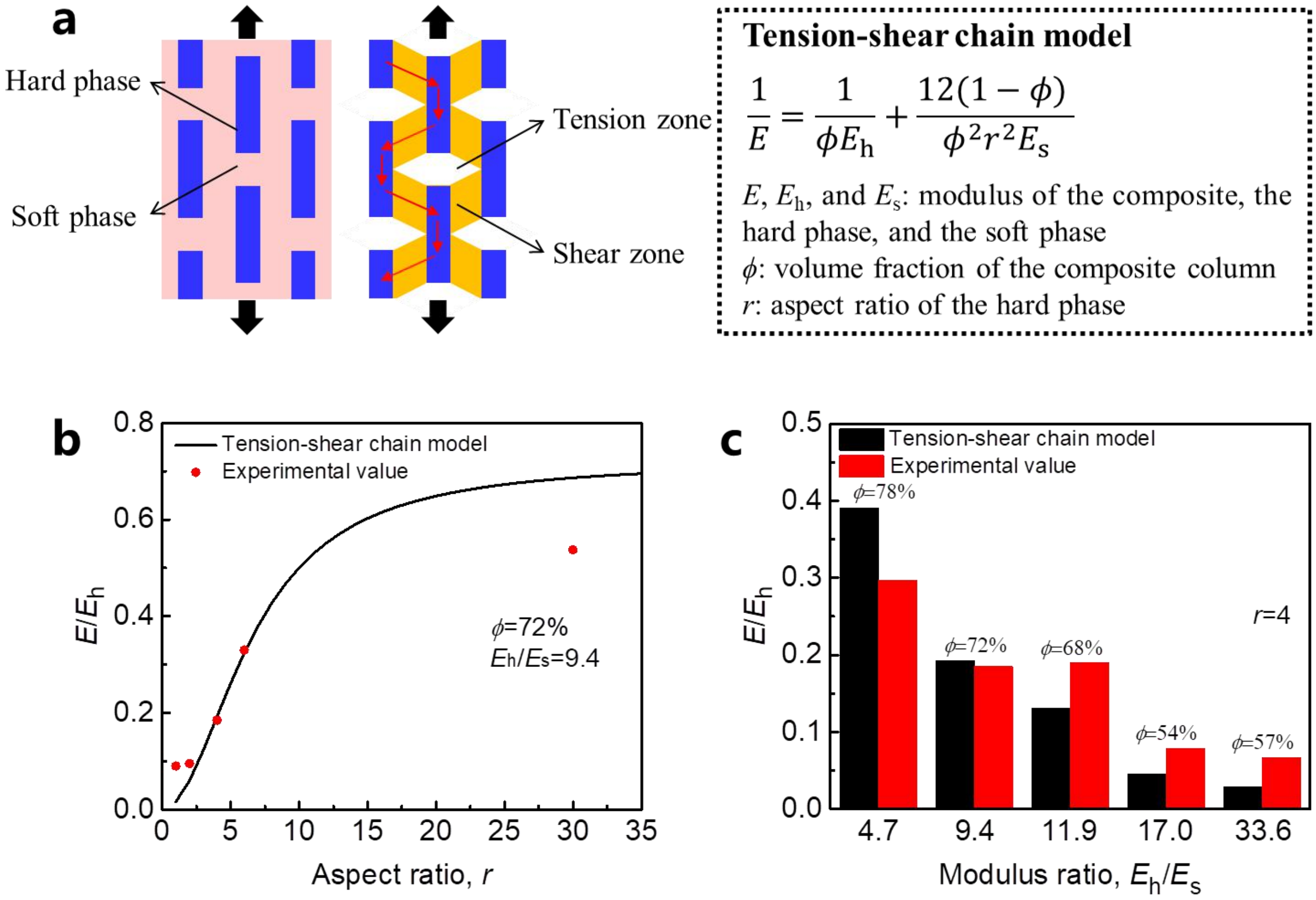

Supplementary Figure 7. Comparison of the modulus of TPNs predicted by the tension-shear chain model and by experimental measurement. a, A tension-shear chain model of TPNs in which the tensile zones of the soft phase are eliminated to emphasize the load transfer within the composite structure. The analytic equation for the modulus of TPNs refers to the previous report[2], including four parameters as E_h, E_s, ϕ, and r. **b,** E/E_h as a function of the aspect ratio r of the hard phase. The experimental values are in good agreement with the theoretical predictions from the tension-shear chain model. Note that the experimental E/E_h at $r = \infty$ is approximately plotted at $r = 30$, because the tension-shear chain model predicts the E/E_h nearly keeps constant when $r \geq 30$. **c,** E/E_h as a function of the modulus ratio E_h/E_s of the hard phase to the soft phase. When changing the crosslinker concentration for the PAMPS network, ϕ and E_h/E_s vary simultaneously, but r keeps constant. The experimental values are close to the theoretical predictions.

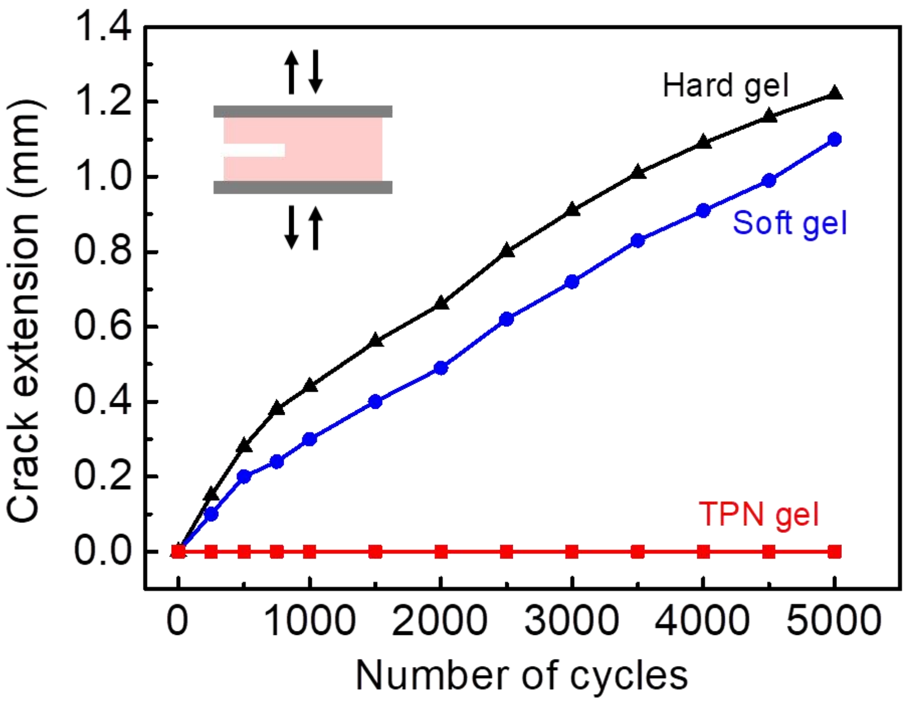

Supplementary Figure 8. Fatigue test for precut samples. The soft, hard, and TPN gel with a rectangular geometry (width 50 mm and height 10 mm) and a 20 mm one-edge crack, were subjected to cyclic loads over 5000 cycles at the maximum strain of 0.5. The loading frequency was fixed at 1 Hz. The crack extension was monitored by a video camera (Nikon 5200).

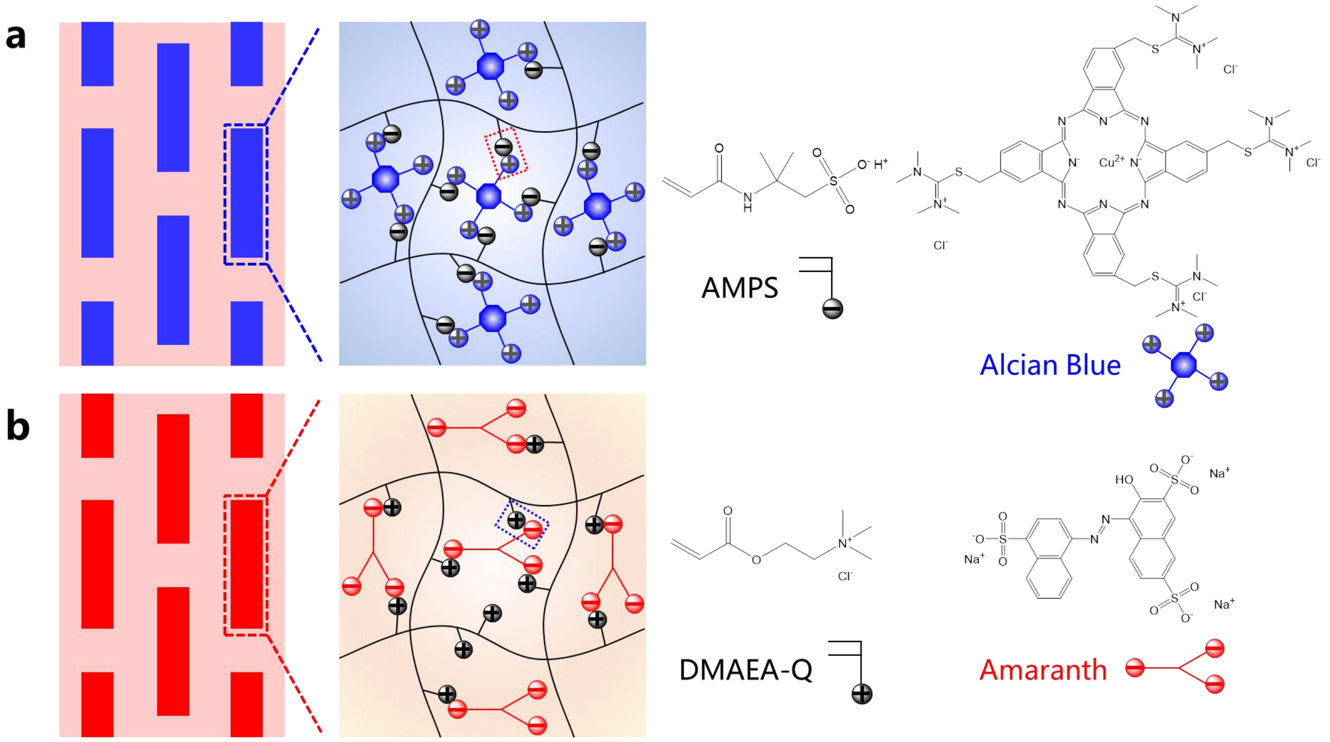

Supplementary Figure 9. Dyeing mechanism of TPNs. a, b, Based on electrostatic attraction, the PAMPS-based gel (a) and the PDMAEA-Q-based gel (b) are selectively dyed by Alcian Blue and Amaranth in the hard phase, respectively.

Supplementary References

1 Liang, S. M. *et al.* Direct Observation on the Surface Fracture of Ultrathin Film Double-Network Hydrogels. *Macromolecules* **44,** 3016-3020 (2011).

2 Ji, B. H. & Gao, H. J. Mechanical properties of nanostructure of biological materials. *J. Mech. Phys. Solids* **52**, 1963-1900 (2004).
