# Supplementary material for: Topoarchitected polymer networks expand the space of material properties
Source: Nat Commun. 2022 Mar 25;13:1622. doi: 10.1038/s41467-022-29245-0 (PMC8956700; doi:10.1038/s41467-022-29245-0)
Supplement: Supplementary file 3 — Description of Additional Supplementary Files [file 41467_2022_29245_MOESM3_ESM.pdf]

Description of Additional Supplementary Files

Supplementary Movie 1: Pure shear test of the precut soft gel

Supplementary Movie 2: Pure shear test of the precut hard gel

Supplementary Movie 3: Pure shear test of the precut TPN gel

Supplementary Movie 4: Tensile test of the uncut TPN gel

Supplementary Movie 5: Peel test of the bilayer TPN gel
